# Physical activity is associated with a lower risk of contracting and dying in infection and sepsis: a Swedish population-based cohort study

**DOI:** 10.1186/s13054-024-04881-8

**Published:** 2024-03-24

**Authors:** Karl Stattin, Mikael Eriksson, Robert Frithiof, Rafael Kawati, Michael Hultström, Miklos Lipcsey

**Affiliations:** 1 Department of Surgical Sciences, Anesthesiology and Intensive Care, Uppsala University, Akademiska Sjukhuset, Ingång 70, 75 185 Uppsala, Sweden; 2 Department of Medical Cell Biology, Unit for Integrative Physiology, Uppsala University, Uppsala, Sweden; 3 Hedenstierna Laboratory, Department of Surgical Sciences, Anesthesiology and Intensive Care, Uppsala University, Uppsala, Sweden

**Keywords:** Sepsis, Mortality, Physical activity, Exercise, Walking, Infection

## Abstract

**Background:**

Sepsis is a condition where the immune response to infection becomes dysregulated and life-threatening. It is not known whether lifestyle factors influence the risk of sepsis. The aim of the present study is to investigate the association between physical activity and the risk of acquiring and dying in infection or sepsis.

**Methods:**

The population-based Swedish Mammography Cohort and Cohort of Swedish Men sent participants lifestyle questionnaires in 1997 and have subsequently followed participants in national Swedish registers, including the National Patient Register, the Swedish Intensive Care Registry and the Cause of Death Register. The risk of contracting infection and sepsis, the risk of intensive care unit admission and the risk of death were estimated using multivariable Cox regression.

**Results:**

Among 64,850 cohort participants, 26,124 individuals suffered at least one episode of infection or sepsis and 4708 individuals died of infection or sepsis during the study period. In adjusted analyses, compared to exercising less than one hour per week, stated exercise one hour per week was associated with lower risk of contracting infection or sepsis, hazard ratio (HR) 0.93 (95% confidence interval (CI) 0.90–0.97), and lower risk of dying in infection or sepsis, HR 0.87 (95% CI 0.80–0.96). Further exercise was associated with even lower risk, and similar patterns were observed for walking. The population-attributable risks of contracting and dying in infection or sepsis for not exercising were 2.6% and 4.5%, respectively.

**Conclusions:**

Exercise and walking demonstrate inverse dose–response associations with both the risk of contracting and dying in infection and sepsis, presenting possible preventative interventions for this critical condition.

**Supplementary Information:**

The online version contains supplementary material available at 10.1186/s13054-024-04881-8.

## Introduction

Sepsis is a condition where the immune response to severe infection becomes dysregulated and life-threatening [1]. Sepsis patients treated in intensive care units (ICUs) have a 30-day mortality of 33% and a one-year mortality of almost 50% [2]. Sepsis is estimated to cause almost one in ten deaths in the USA [3]. However, most patients that die in sepsis do not die due to the infection itself, but premorbid conditions and physiological reserves play an important role [4]. Physical activity lowers the risk of many noncommunicable diseases [5] and affects immune function [6] and cardiopulmonary capacity [7] and may thus reduce the risk of contracting sepsis and dying in sepsis. However, the majority of US adults are physically inactive [8].

The association between physical activity and sepsis is sparsely investigated. Results are conflicting [9], but previous studies have indicated that physical activity may reduce the risk of contracting sepsis [10] and dying in sepsis [11]. In the positive studies, no dose–response relations were observed, but rather a threshold effect was observed where inactive individuals were at higher risk [10, 11]. Prior studies have not compared different intensities of physical activity, and no study has investigated the full spectrum of disease, i.e., the risk of contracting sepsis, the risk of intensive care admission and the risk of dying in sepsis.

The aim of this study is thus to investigate whether physical activity; be it low-intensity walking; intense exercise; physical activity at work; or sedentary behavior such as reading or watching television; is associated with contracting infection or sepsis, ICU admission or dying due to infection or sepsis and to elucidate the shape of the associations.

## Methods

This study was approved by the National Ethical Review Agency, Stockholm, Sweden (DNR 2022-05751-01). Informed consent was obtained from all participants on inclusion into the cohorts. The Helsinki Declaration was followed, as was the STROBE statement [12].

### Availability of data and materials

Due to the sensitive nature of the collected data, data cannot be publicly shared. However, researchers with a valid ethical approval may contact the steering committee of the Swedish Infrastructure for Medical Population-based Life-course and Environmental Research (SIMPLER) [13] to access data.

### Participants

Participants were drawn from the population-based Swedish Mammography Cohort (SMC) and Cohort of Swedish Men (COSM). The SMC was started in 1987 by inviting all women born 1914–1948 living in Uppsala county and all women born 1917–1948 living in Västmanland county to answer self-administered lifestyle questionnaires. This first questionnaire had a participation rate of 74%. A second more detailed questionnaire was administered in 1997 and was answered by 70% (*n* = 39,277) of the original cohort. COSM was started in 1997 by sending a questionnaire similar to the one used in SMC to all men born 1918–1952 living in Västmanland and Örebro counties. The response rate was 49% (*n* = 48,850). The SMC and COSM are representative of the Swedish population [14, 15]. Participants have been linked to the National Patient Register, the Swedish Intensive Care Registry (SIR) and the Cause of Death Register, using the unique Personal Identification Number issued to all residents in Sweden.

All participants having entered an incorrect personal identification number, that died before January 1st, 1998, or were diagnosed with cancer (except nonmelanoma skin cancer) were excluded, leaving 38,984 women aged 48 to 83 years and 45,906 men aged 45 to 79 at baseline in the final cohort (Additional file 1).

### Physical activity and covariates

Physical activity was ascertained through self-administered lifestyle questionnaires administered in 1997. Questions inquired about time spent on leisure exercise (< 1 h/week, 1 h/week, 2–3 h/week, 4–5 h/week or > 5 h/week), time spent walking or bicycling (hardly ever, < 20 min/day, 20–40 min/day, 40–60 min/day, 60–90 min/day or > 90 min/day, the highest two levels were collapsed for analysis), physical activity at work (mostly sitting, sitting half of the time, most standing, mostly walking with little carrying, mostly walking with considerable carrying, heavy manual labor, levels were collapsed into: sitting, sitting half of the time, standing and walking, lifting), reading or watching television (< 1 h/day, 1–2 h/day, 3–4 h/day, 5–6 h/day, > 6 h/day, the highest two levels were combined) and household work (< 1 h/day, 1–2 h/day, 3–4 h/day, 5–6 h/day, 7–8 h/day, > 8 h/day, the highest three levels were combined). The measures of physical activity have been validated against 7-day activity records with correlations of circa 0.4 [16, 17] and against accelerometer data with correlation of 0.38 [17], suggesting acceptable validity.

Covariates were selected using a directed acyclic graph (DAG)-based method [18]. All covariates were collected from the questionnaires, except comorbidities which were collected from the National Swedish Patient Register, which has near-complete coverage and high validity [19].

### Outcome

Incident cases of infection or sepsis were collected from the Swedish National Patient Register until 31th December 2021. First occurrence of infection or sepsis was grouped and defined according to the International Statistical Classification of Diseases and Related Health Problems (ICD-10) diagnosis codes other sepsis (A40, A41, A32, A48, A49, B95, B96 D65, T802, R65.1, R57.2), abdominal infection (K35, K57.0, K57.2, K57.4, K57.8, K63.0, K63.1, K65, K80.0, K80.1, K80.3, K80.4, K81, K83.0, K85), urogenital infection (N10, N12, N13.6, N39.0, N70), soft tissue infection (M00, M01, M72.6, A46), pneumonia (J13, J14, J15, J16, J18, J85, J86), endocarditis (I33, I39), tuberculosis (A15, A16, A17, A18, A19) and central nervous system infection (A39, G00, G01, G02, G03.9, G05.0, G06, G07.9), see Additional file 2. ICU admission, physiological derangement at admission (classified using the Simplified Acute Physiology Score, SAPS3 [20]) and organ-replacement therapy were acquired from the Swedish Intensive Care Registry [21]. Death due to infection or sepsis was drawn from the Cause of Death Register, using the same ICD-10 codes.

### Statistical analysis

Using Cox proportional hazards regression with attained age as timescale, hazard ratio (HR) and 95% confidence intervals (CI) were calculated for first occurrence of infection or sepsis, ICU admission, and death due to infection or sepsis. Participants contributed person-time at risk from baseline (January 1st 1998) until infection or sepsis, death or end of follow-up (December 31st, 2021), whichever occurred first. Exposures of interest were exercise; walking; physical activity at work; reading or watching television and household physical activity. The lowest level was used as reference. Estimates were first calculated in a crude model adjusted only for age (as timescale) and sex, and subsequently in a fully adjusted model, including age (as timescale), sex (man/woman), marital status (cohabiting/living alone), education (≤ 9 years/9–12 years/ > 12 years/other, such as vocational), smoking status (current/former/never), alcohol consumption (g/day, continuous) and Charlson’s weighted comorbidity index [22] (continuous). To test whether potential associations are linear, adjusted models using exposures as continuous variables and as restricted cubic splines with the median as reference and knots placed at the 10th and 90th percentile [23] were compared using likelihood ratio (LR) tests. Log–log plots were used to test the proportional hazards assumption. The population-attributable fraction (PAF) for contracting and dying in infection or sepsis, respectively, was calculated as:

$${\text{PAF}}=Pc*(1-\frac{1}{{\text{HR}}})$$

where *Pc* is the proportion of exposure among cases and HR is the adjusted hazard ratio [24]. For this analysis, the lowest level of exercise (to approximate inactivity) was chosen as exposure, and all other levels of exercise were collapsed and treated as unexposed.

The main analysis was performed using complete-case analysis. The proportion of missing was 13.8% for physical activity at work, 11.0% for exercise and 10.0% for household work. The proportion of missing was < 10% for all other variables. For the main analysis of exercise, 64,850 participants had all information necessary for inclusion (see Additional file 1: Cohort flowchart).

Sensitivity analyses were performed to test the robustness of results. Two sensitivity analyses were performed to attempt to further adjust for confounding by previous health (i.e., if previous illness or poor health leads to an individual being less physically active): first the main analysis was further adjusted for self-rated health, and second, an analysis with a washout period of three years was performed, where participants became at risk January 1, 2001. To account for the possibility of nonlinear effects in confounding by comorbidity, a sensitivity analysis was performed where Charlson’s weighted comorbidity index was included as a categorical variable (0/1/ ≥ 2). The main analysis of death included infection and sepsis both as *underlying* and *contributing* causes of death in case of multiple causes of death. A sensitivity analysis was performed where only the *underlying* cause of death was considered. The main analysis was repeated, but with December 31st, 2019, as the end of follow-up, to exclude Covid-19 pandemic years. To assess whether observed associations were mediated by body mass index (BMI, weight in kilograms divided by length in meters squared), a sensitivity analysis was performed adjusting for BMI divided into categories (< 20/ ≥ 20 to < 25/ ≥ 25 to < 30/ ≥ 30). Analyses were performed for each diagnosis group separately. To check whether associations were similar in smokers and nonsmokers, the main analysis was repeated stratified for smoking status.

Descriptive statistics are presented as median (interquartile range, IQR). All analyses were performed in Stata 15.1 (Stata Corp., College station, Texas, USA).

## Results

Characteristics of cohort participants available for the main analysis are presented in Table 1, and characteristics by level of physical activity are presented in Additional file 3. The median age at baseline was 60 years (IQR 53–68) and 58.4% were men.

**Table 1:** Participant characteristics

	Participants
Total number	64,850
Age, years (IQR)	60.0 (53.0–68.0)
Alcohol, g/day (IQR)	8.7 (2.5–19.2)
Women, *n* (%)	26,967 (41.6)
Marital status, *n* (%)	
Cohabiting	52,197 (80.5)
Living alone	12,653 (19.5)
Smoking, *n* (%)	
Never	26,870 (41.4)
Former	15,501 (23.9)
Current	22,479 (34.7)
Education, *n* (%)	
< 9 years	23,488 (36.2)
9–12 years	4281 (6.6)
> 12 years	11,510 (17.7)
Other	25,571 (39.4)
Charlson’s weighted comorbidity index, *n* (%)	
0	53,909 (83.1)
1	6686 (10.3)
2	3084 (4.8)
≥ 3	1171 (1.8)
Exercise, *n* (%)	
< 1 h/week	13,595 (21.0)
1 h/week	13,570 (20.9)
2–3 h/week	20,944 (32.3)
4–5 h/week	8023 (12.4)
> 5 h/week	8718 (13.4)
Walking, *n* (%)	
Hardly ever	7726 (12.2)
< 20 min/day	14,029 (22.2)
20–40 min/day	19,918 (31.6)
40–60 min/day	10,581 (16.8)
> 60 min/day	10,870 (17.2)
Physical activity at work, *n* (%)	
Mostly sitting	13,042 (21.7)
Sitting half time	19,361 (32.2)
Standing, walking	20,350 (33.9)
Lifting	7291 (12.1)
Reading/TV, *n* (%)	
< 1 h/day	7535 (11.7)
1–2 h/day	29,907 (46.6)
3–4 h/day	23,012 (35.8)
> 5 h/day	3765 (5.9)
Household physical activity, *n* (%)	
< 1 h/day	16,292 (26.0)
1–2 h/day	25,169 (40.2)
3–4 h/day	13,626 (21.8)
> 5 h/day	7478 (12.0)

*IQR* interquartile range

In the analysis of exercise and occurrence of infection or sepsis, 64,849 individuals contributed 1,264,850 person-years at risk (PYAR) and 26,124 suffered a first episode of infection or sepsis, of which 5153 (19.7%) was other sepsis, 4187 (16.0%) abdominal, 8782 (33.6%) urogenital, 2461 (9.4%) soft tissue, 7776 (29.8%) pneumonia, 113 (0.4%) endocarditis, 73 (0.3%) tuberculosis and 191 (0.7%) central nervous system infections. In the analysis of exercise and death, 64,850 individuals contributed 1,250,150 PYAR, during which 4708 individuals died of infection or sepsis, where the underlying or contributing cause of death was other sepsis in 1155 individuals (24.5%), abdominal in 444 (9.4%) individuals, urogenital in 483 (10.3%) individuals, soft tissue in 67 (1.4%) individuals, pneumonia in 2964 (63.0%) individuals, endocarditis in 27 (0.6%) individuals, tuberculosis in 9 (0.2%) individuals and central nervous system infection in 18 (0.4%) individuals. Note that multiple infections may be coded as admission diagnoses and classified as contributing causes of death, and that percentages therefore add up to more than 100%.

### Risk of infection or sepsis

**Fig. 1:**
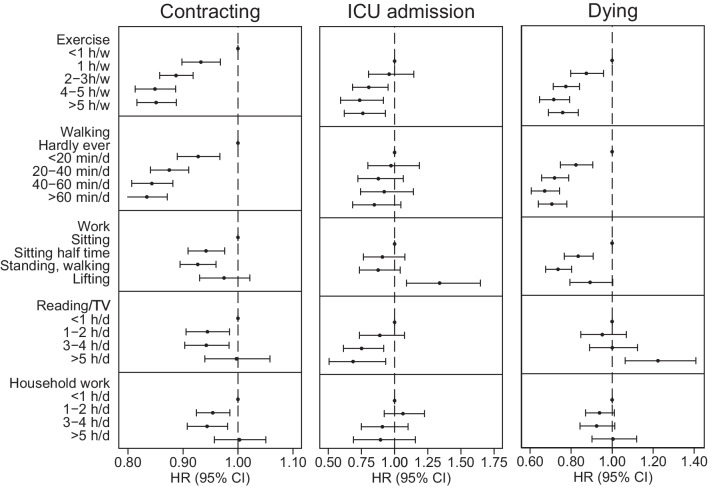
Risk of infection, sepsis, intensive care unit admission and death. Hazard ratio (HR) and 95% confidence interval (CI) of infection and sepsis, intensive care unit admission and death in infection and sepsis, adjusted for age (as timescale), sex, marital status, education, smoking status, alcohol consumption and Charlson’s weighted comorbidity index

Physical activity was associated with a lower risk of contracting a infection or sepsis (Fig. 1, Additional file 4: Table 3). Compared to exercising < 1 h per week, exercising 1 h per week was associated with HR 0.93 (95% CI 0.90–0.97), with progressively lower risk with higher levels of exercise. Similarly, compared to hardly ever walking, walking < 20 min per day was associated with lower risk of infection or sepsis, HR 0.93 (95% CI 0.89–0.97), with further lower risk observed with more walking. Having a physically active work was also associated with lower risk, but no dose–response relation was observed. Reading/watching television and household work were associated with marginally lower risk of infection or sepsis. LR tests demonstrated departure from linearity for all types of physical activity. The population-attributable fraction of not exercising for contracting infection or sepsis was 2.6%. Sensitivity analyses adjusted for self-rated health showed similar associations as the main analysis (Additional file 5: Fig. 2), as did analyses restricted to commence three years after baseline (Additional file 6: Fig. 3), analysis adjusting for Charlson’s weighted comorbidity index as a categorical variable (Additional file 7: Fig. 5), analysis restricted to pre-covid-19 years (Additional file 8: Fig. 6) and analyses adjusted for BMI (Additional file 9: Fig. 7). Sensitivity analyses investing each group of infections separately had low power, but the larger groups showed similar results as the main analysis (Additional file 10: Fig. 8). Associations were similar in never, former and current smokers (Additional file 11: Fig. 9).

### ICU admission

Participant characteristics at ICU admission are displayed in Table 2. During follow-up, 1078 individuals were admitted to an ICU due to infection, of which 388 were treated with mechanical ventilation and 97 with renal replacement therapy. Statistical power was thus low, but compared with not exercising, exercising for two to three hours per week was associated with lower risk of ICU admission, HR 0.80 (95% CI 0.68–0.95) (Fig. 1, Additional file 4: Table 3). LR tests indicated linear relations for exercise, walking, reading/TV and household physical activity.

**Table 2:** Characteristics of participants admitted to an intensive care unit

	Participants admitted to an ICU
Total number	1078
Age, years (IQR)	76.0 (71.0–81.0)
Alcohol, g/day (IQR)	10.6 (2.9–22.7)
SAPS3 (IQR)	67.0 (59.0–76.0)
Women, *n* (%)	255 (23.7)
Marital status, *n* (%)	
Cohabiting	866 (80.3)
Living alone	212 (19.7)
Smoking, *n* (%)	
Never	334 (31.0)
Former	353 (32.7)
Current	391 (36.3)
Education, *n* (%)	
< 9 years	419 (38.9)
9–12 years	75 (7.0)
> 12 years	159 (14.7)
Other	425 (39.4)
Charlson’s weighted comorbidity index, *n* (%)	
0	857 (79.5)
1	132 (12.2)
2	69 (6.4)
≥ 3	20 (1.9)
Exercise, *n* (%)	
< 1 h/week	260 (24.1)
1 h/week	234 (21.7)
2–3 h/week	316 (29.3)
4–5 h/week	120 (11.1)
> 5 h/week	148 (13.7)
Walking, *n* (%)	
Hardly ever	153 (14.5)
< 20 min/day	255 (24.2)
20–40 min/day	296 (28.1)
40–60 min/day	167 (15.9)
> 60 min/day	182 (17.3)
Physical activity at work, *n* (%)	
Mostly sitting	220 (21.9)
Sitting half time	314 (31.2)
Standing, walking	312 (31.0)
Lifting	159 (15.8)
Reading/TV, *n* (%)	
< 1 h/day	132 (12.4)
1–2 h/day	508 (47.6)
3–4 h/day	368 (34.5)
> 5 h/day	59 (5.5)
Household physical activity, *n* (%)	
< 1 h/day	319 (30.4)
1–2 h/day	470 (44.7)
3–4 h/day	177 (16.8)
> 5 h/day	85 (8.1)
Mechanical ventilation, *n* (%)	388 (36.0)
Noninvasive ventilation, *n* (%)	276 (25.6)
High-flow oxygen, *n* (%)	189 (17.5)
Renal replacement therapy, *n* (%)	97 (9.0)
ICU mortality, *n* (%)	204 (18.9)

*ICU* intensive care unit, *IQR* interquartile range, *SAPS3* Simplified Acute Physiology Score 3

### Risk of death

Exercise, walking and physical activity at work were associated with lower risk of dying in infection or sepsis (Fig. 1, Additional file 4: Table 3). Compared to exercising < 1 h per week, exercising 1 h per week was associated with lower risk of dying in infection or sepsis, HR 0.87 (95% CI 0.80–0.96), with indications of a dose–response relationship. A similar pattern was observed for walking, whereas again no dose–response relationship was observed for physical activity at work. The highest level of reading or watching television was associated with a higher risk of dying in infection or sepsis, HR 1.24 (95% CI 1.08–1.43) compared to reading or watching television < 1 h per day. Household work was not associated with dying in infection or sepsis. No exposure demonstrated linear association with mortality in LR tests. The population-attributable fraction of not exercising for dying in infection or sepsis was 4.5%. Analyses adjusted for self-rated health (Additional file 5: Fig. 2), starting time at risk three years after baseline (Additional file 6: Fig. 3), adjusting for Charlson’s weighted comorbidity index as a categorical variable (Additional file 12: Fig. 4), only including underlying cause of death (Additional file 7: Fig. 5), analyses restricted to years prior to covid-19 (Additional file 8: Fig. 6) and analyses adjusted for BMI (Additional file 9: Fig. 7) all showed similar results as the main analysis. Sensitivity analyses separating infections by group had low power, but indicated results similar as the main analysis (Additional file 10: Fig. 8). Associations were similar in never, former and current smokers (Additional file 11: Fig. 9).

## Discussion

In this large population-based cohort study, many types of physical activity were associated with lower risk of contracting infection or sepsis and lower risk of dying in infection or sepsis. Leisure physical activity, i.e., exercise and walking, demonstrated dose–response relationships with the risk of contracting and dying in infection or sepsis, whereas physical activity at work demonstrated a threshold effect, where sitting work was associated with higher risks than other types of work. Household work was not associated with lower risk of dying in sepsis, and high levels of reading or watching television was associated with a higher risk of dying in infection or sepsis. Analyses of ICU care were hampered by low power, but exercising may be associated with lower risk of ICU admission. The population-attributable fraction of inactivity for death in infection or sepsis was 4.5%, which may be compared to previous studies indicating PAF of inactivity of 8.8% for all-cause mortality [25] and 14% for mortality in coronary heart disease [26]. Previously, sepsis prevention has mainly prompted hygiene and early detection of infection [27–29], but this study indicates that physical activity may decrease an individual’s risk of contracting and dying in infection and sepsis.

Whereas the association between physical activity and cardiovascular disease is established [30], the association with sepsis is considerably less well-studied. Wang et al. found that participating in no exercise was associated with higher risk of sepsis compared to exercising ≥ 4 times per week, whereas there was no difference in risk among individuals exercising 1–3 times per week or ≥ 4 times per week. They found no association between watching television and sepsis [10]. In a cohort of active walkers and runners, Williams found that both walking and running were associated with lower risk of dying in sepsis [11]. In unadjusted analyses, Baik et al. found a dose–response trend where physical activity was associated with lower risk of community-acquired pneumonia in women, but had few cases and only studied pneumonia and in adjusted analyses, the association was attenuated [9]. Hence, contrary to Baik et al., both Wang et al. and Williams found potential threshold effects, where no physical activity was associated with higher risk compared to any level of physical activity [10, 11]. Concurrent with previous studies, the greatest risk difference in this study was observed between sedentary individuals and individuals with any level of physical activity, but we found further lower risk with increasing levels of activity up to 5 h per week. This may be due to the larger study size with more cases in the present study. Surprisingly, a modest level of reading or watching television was associated with minor reductions in the risk of contracting sepsis, whereas high levels of reading or watching television was associated with a higher risk of dying in infection or sepsis. This may be due to unmeasured confounding by healthy habits associated with reading. Analyses of ICU admission demonstrated wide confidence intervals. This is partly due to low power, but may also be due to conflicting underlying associations: as exercise was associated with a lower risk of contracting sepsis, individuals that exercise have a lower risk of needing intensive care treatment. However, if they do contract sepsis and need intensive care treatment, it is likely that they will be admitted. Unfortunately, we do not have prerequisite data to explore potential underlying associations.

There are several hypotheses as to possible mechanisms underlying a potential association between physical activity and sepsis. Whereas a weak immune system may predispose to contracting and dying in infection, an immunological overreaction to infection leads to sepsis; meaning the immune response to infection needs to be balanced. Physical activity affects immune function [6] and could thus potentially influence this balance. Further, most patients that die in sepsis do not die due to the infection or sepsis itself but due to an interplay between the acute condition and preexisting disease and functional status [4]—factors that physical activity have been shown to affect [7].

Strengths of the present study include exposure information collected using validated questionnaires administered to large population-based cohorts representative of the Swedish population with long and virtually complete follow-up in national registers. The main limitation is the use of self-reported physical activity from a single baseline questionnaire. However, self-report is likely to overestimate physical activity [31], which would attenuate the results. Observational studies always suffer from the possibility of unmeasured or residual confounding even if known confounders are adjusted for in multivariable regression. In order to strengthen the evidence for a causal association between physical activity and sepsis, we plan to examine the association using Mendelian randomization and twin methodology in the future. Not all patients in the present cohort were diagnosed with sepsis per se, but infection was used as a surrogate. Further, using sepsis as defined by ICD codes in registers has weaknesses [32].

In conclusion, physical activity is associated with lower risk of contracting and dying in infection or sepsis, with a non-negligible population-attributable risk. Further, engaging in exercise and walking is associated with a lower risk of contracting and dying in infection and sepsis in a dose-dependent manner. If these results are replicated, this suggests that increasing physical activity may present an opportunity to reduce the risks of sepsis.

### Supplementary Information

**Additional file 1: Fig. 1.** Cohort flowchart.

**Additional file 2: Table 1.** International Classification of Diseases and Related Health Problems (ICD-10) codes for included infectious diseases and sepsis.

**Additional file 3: Table 2.** Participant characteristics by level of physical activity.

**Additional file 4: Table 3.** Number of individuals, number of cases, person-years at risk (PYAR), hazard ratio (HR) and 95% confidence interval (CI) for crude and adjusted analyses of the risk of acquiring infection or sepsis, of intensive care unit admission, and of dying in infection or sepsis. Crude analyses adjusted for age (as timescale) and sex, adjusted analyses adjusted for age (as timescale), sex, marital status, education, smoking status, alcohol consumption and Charlson’s weighted comorbidity index.

**Additional file 5: Fig. 2.** Hazard ratio (HR) and 95% confidence interval (CI) of infection and sepsis and death in infection and sepsis, adjusted for self-rated health in addition to age (as timescale), sex, marital status, education, smoking status, alcohol consumption and Charlson’s weighted comorbidity index.

**Additional file 6: Fig. 3.** Hazard ratio (HR) and 95% confidence interval (CI) of infection and sepsis and death in infection and sepsis with January 1st 2001 as the start of follow-up in order to include a three-year period of washout, adjusted for age (as timescale), sex, marital status, education, smoking status, alcohol consumption and Charlson’s weighted comorbidity index.

**Additional file 7: Fig. 5.** Hazard ratio (HR) and 95% confidence interval (CI) of dying in infection and sepsis, where only the underlying cause of death is considered, adjusted for age (as timescale), sex, marital status, education, smoking status, alcohol consumption and Charlson’s weighted comorbidity index.

**Additional file 8: Fig. 6.** Hazard ratio (HR) and 95% confidence interval (CI) of infection and sepsis and death in infection and sepsis, adjusted for age (as timescale), sex, marital status, education, smoking status, alcohol consumption and Charlson’s weighted comorbidity index with December 31st 2019 as end of follow-up.

**Additional file 9: Fig. 7.** Hazard ratio (HR) and 95% confidence interval (CI) of infection and sepsis and death in infection and sepsis, adjusted for age (as timescale), sex, marital status, education, smoking status, alcohol consumption, Charlson’s weighted comorbidity index and BMI.

**Additional file 10: Fig. 8.** Hazard ratio (HR) and 95% confidence interval (CI) of contracting and dying in other sepsis, abdominal, urogenital, soft tissue, pneumonia, endocarditis, tuberculosis, and central nervous system infections, adjusted for age (as timescale), sex, marital status, education, smoking status, alcohol consumption and Charlson’s weighted comorbidity index.

**Additional file 11: Fig. 9.** Hazard ratio (HR) and 95% confidence interval (CI) of infection and sepsis and death in infection and sepsis stratified on smoking status (never smokers: black circles; former smokers: dark gray triangles; current smokers: light gray diamonds), adjusted for age (as timescale), sex, marital status, education, alcohol consumption and Charlson’s weighted comorbidity index.

**Additional file 12: Fig. 4.** Hazard ratio (HR) and 95% confidence interval (CI) of infection and sepsis and death in infection and sepsis, adjusted for age (as timescale), sex, marital status, education, smoking status, alcohol consumption and Charlson’s weighted comorbidity index as a categorical variable.

## Data Availability

Due to the sensitive nature of the collected data, data cannot be publicly shared. However, researchers with a valid ethical approval may contact the steering committee of the Swedish Infrastructure for Medical Population-based Life-course and Environmental Research (SIMPLER) [13] to access data.

## Abbreviations

BMI: Body mass index
CI: Confidence interval
COSM: Cohort of Swedish Men
DAG: Directed acyclic graph
HR: Hazard ratio
ICU: Intensive care unit
ICD: International Statinstical Classification of Diseases and Related Health Problems
IQR: Interquartile range
PYAR: Person-years at risk
SIMPLER: Swedish Infrastructure for Medical Population-based Life-course and Environmental Research
SIR: Swedish Intensive Care Registry
SMC: Swedish Mammography Cohort
