# Supplementary figures and images for: Physical activity is associated with a lower risk of contracting and dying in infection and sepsis: a Swedish population-based cohort study
Source: Crit Care. 2024 Mar 24;28:98. doi: 10.1186/s13054-024-04881-8 (PMC10962192; doi:10.1186/s13054-024-04881-8)

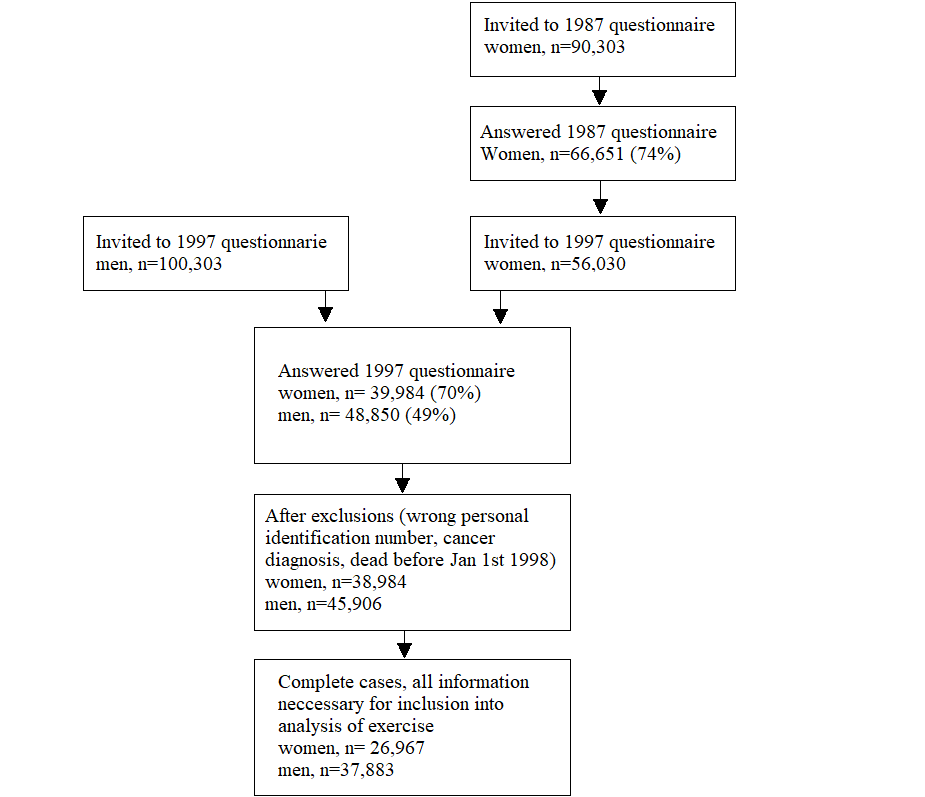

Supplement: Supplementary file 1 — Additional file 1: Fig. 1. Cohort flowchart. [file 13054_2024_4881_MOESM1_ESM.png]

## Contracting

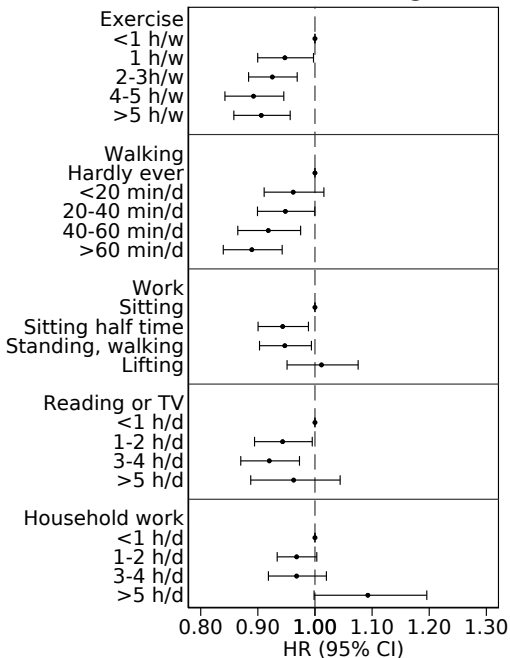

## Dying

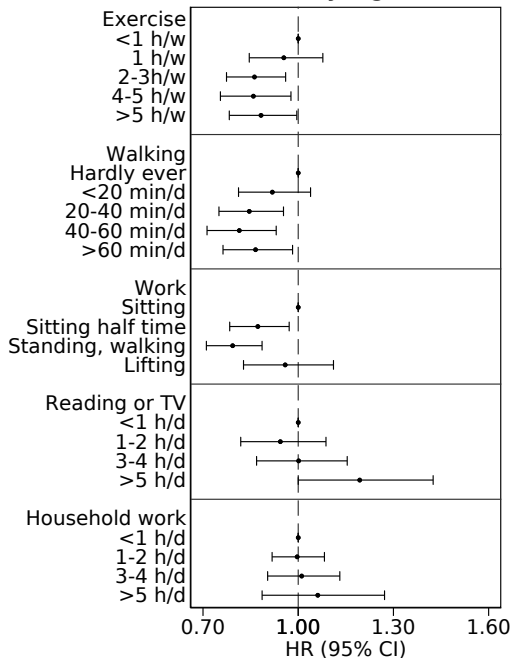

Supplement: Supplementary file 5 — Additional file 5: Fig. 2. Hazard ratio (HR) and 95% confidence interval (CI) of infection and sepsis and death in infection and sepsis, adjusted for self-rated health in addition to age (as timescale), sex, marital status, education, smoking status, alcohol consumption and Charlson’s weighted comorbidity index. [file 13054_2024_4881_MOESM5_ESM.pdf]

## Contracting

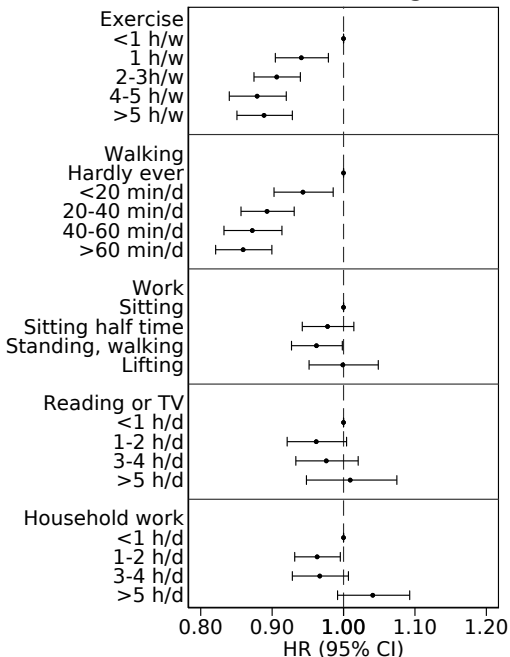

## Dying

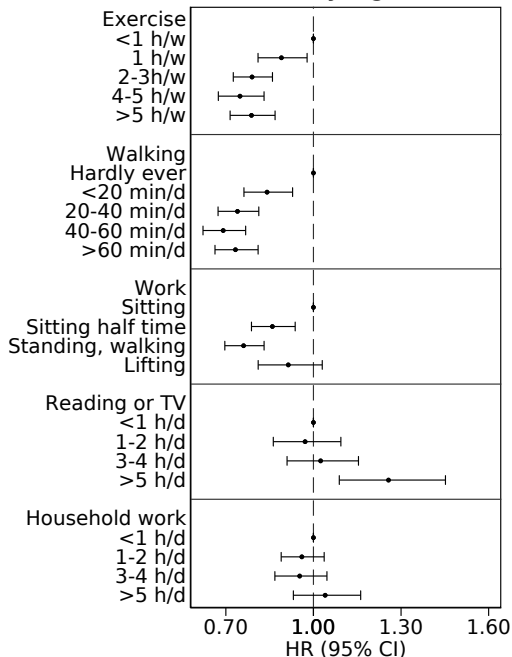

Supplement: Supplementary file 6 — Additional file 6: Fig. 3. Hazard ratio (HR) and 95% confidence interval (CI) of infection and sepsis and death in infection and sepsis with January 1st 2001 as the start of follow-up in order to include a three-year period of washout, adjusted for age (as timescale), sex, marital status, education, smoking status, alcohol consumption and Charlson’s weighted comorbidity index. [file 13054_2024_4881_MOESM6_ESM.pdf]

# Underlying cause of death

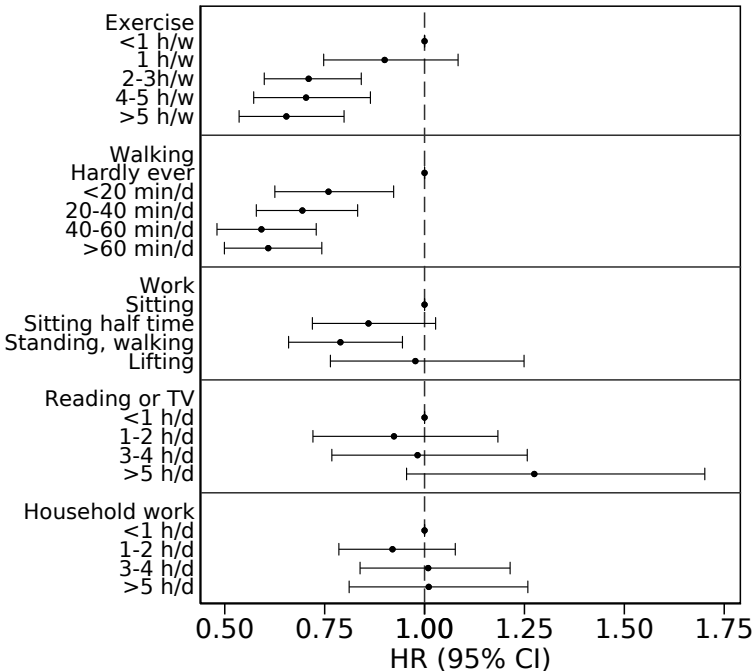

Supplement: Supplementary file 7 — Additional file 7: Fig. 5. Hazard ratio (HR) and 95% confidence interval (CI) of dying in infection and sepsis, where only the underlying cause of death is considered, adjusted for age (as timescale), sex, marital status, education, smoking status, alcohol consumption and Charlson’s weighted comorbidity index. [file 13054_2024_4881_MOESM7_ESM.pdf]

# Covid years excluded, end 31st Dec 2019

## Contracting infection/sepsis:

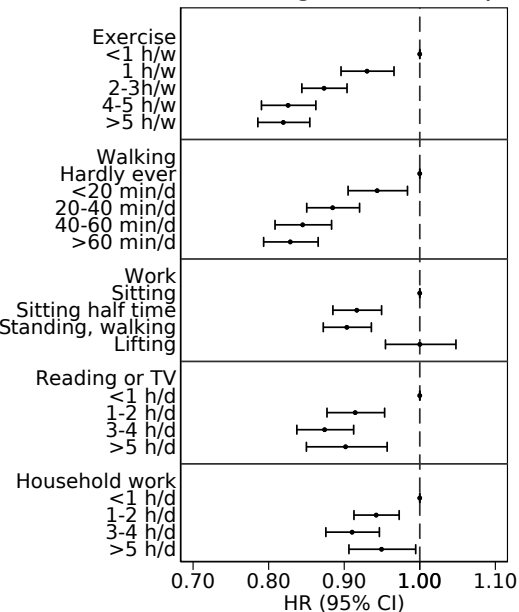

## Dying in infection/sepsis

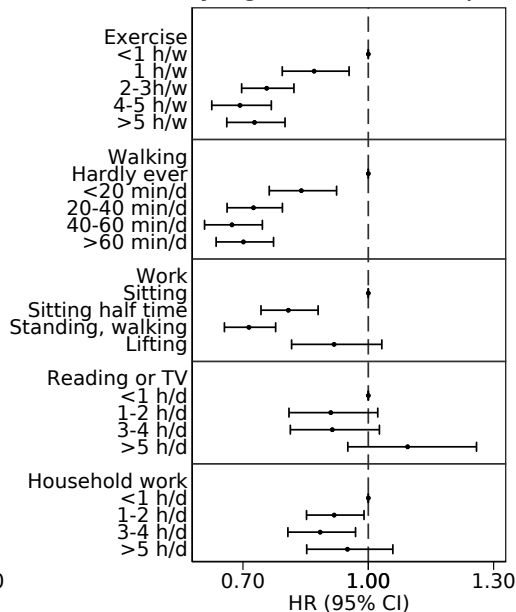

Supplement: Supplementary file 8 — Additional file 8: Fig. 6. Hazard ratio (HR) and 95% confidence interval (CI) of infection and sepsis and death in infection and sepsis, adjusted for age (as timescale), sex, marital status, education, smoking status, alcohol consumption and Charlson’s weighted comorbidity index with December 31st 2019 as end of follow-up. [file 13054_2024_4881_MOESM8_ESM.pdf]

## Contracting

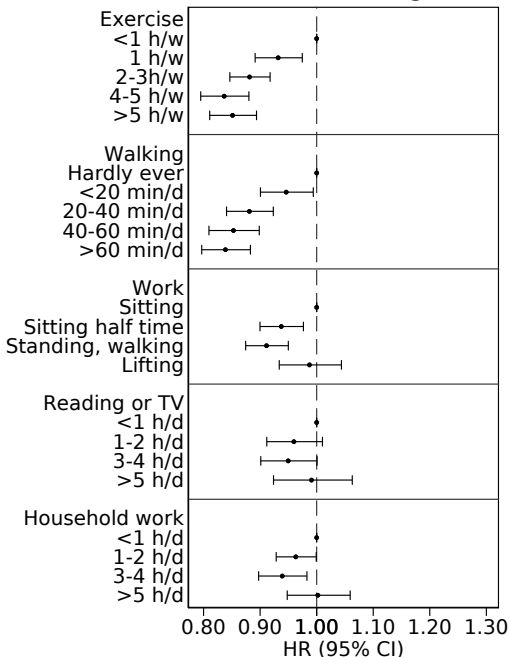

## Dying

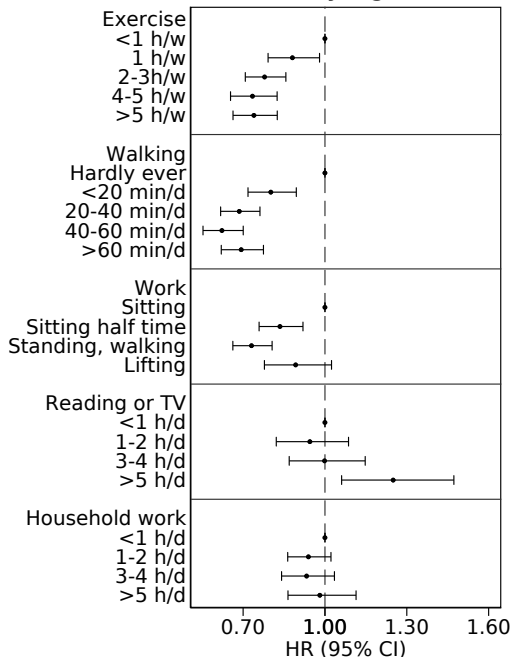

Supplement: Supplementary file 9 — Additional file 9: Fig. 7. Hazard ratio (HR) and 95% confidence interval (CI) of infection and sepsis and death in infection and sepsis, adjusted for age (as timescale), sex, marital status, education, smoking status, alcohol consumption, Charlson’s weighted comorbidity index and BMI. [file 13054_2024_4881_MOESM9_ESM.pdf]

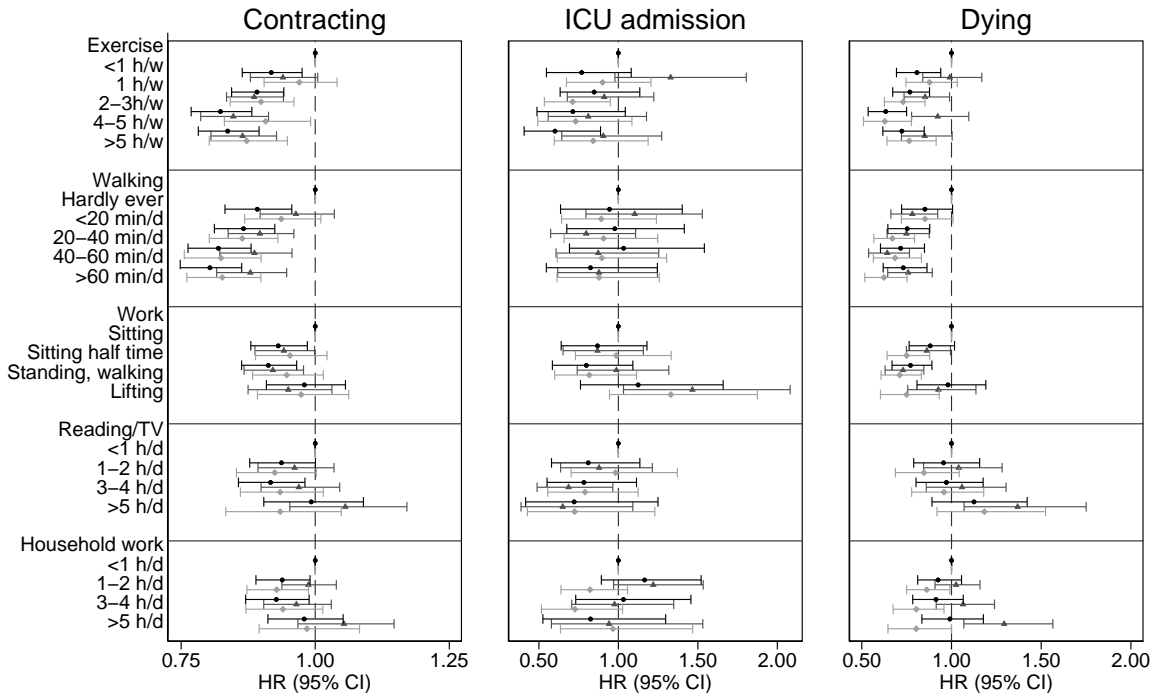

Supplement: Supplementary file 11 — Additional file 11: Fig. 9. Hazard ratio (HR) and 95% confidence interval (CI) of infection and sepsis and death in infection and sepsis stratified on smoking status (never smokers: black circles; former smokers: dark gray triangles; current smokers: light gray diamonds), adjusted for age (as timescale), sex, marital status, education, alcohol consumption and Charlson’s weighted comorbidity index. [file 13054_2024_4881_MOESM11_ESM.pdf]

## Contracting

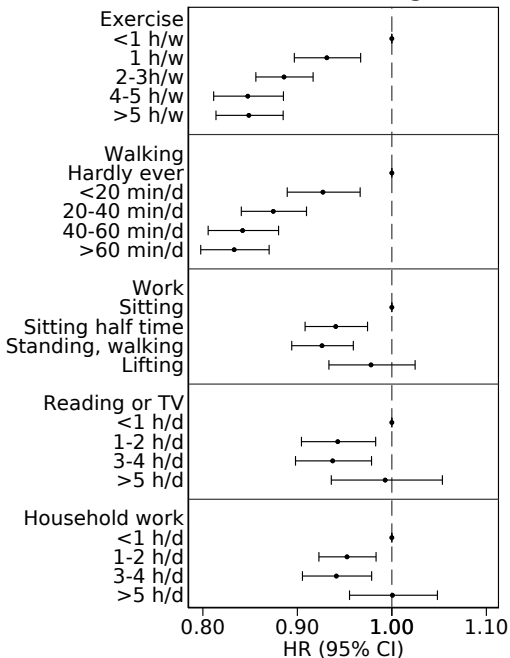

## Dying

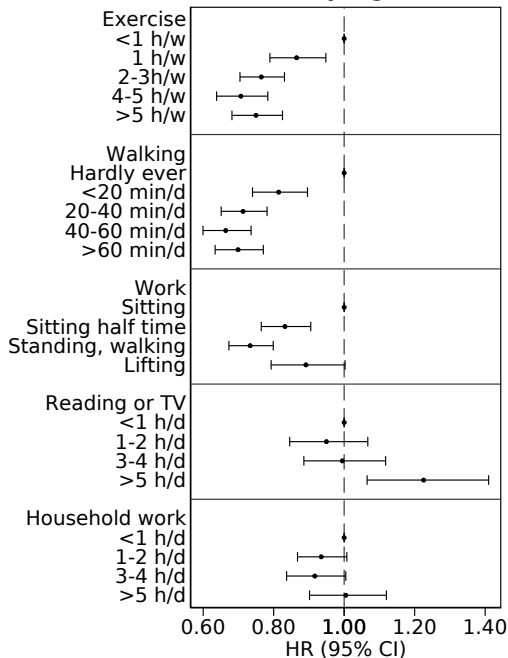

Supplement: Supplementary file 12 — Additional file 12: Fig. 4. Hazard ratio (HR) and 95% confidence interval (CI) of infection and sepsis and death in infection and sepsis, adjusted for age (as timescale), sex, marital status, education, smoking status, alcohol consumption and Charlson’s weighted comorbidity index as a categorical variable. [file 13054_2024_4881_MOESM12_ESM.pdf]
