# Supplementary material for: Physical activity is associated with a lower risk of contracting and dying in infection and sepsis: a Swedish population-based cohort study
Source: Crit Care. 2024 Mar 24;28:98. doi: 10.1186/s13054-024-04881-8 (PMC10962192; doi:10.1186/s13054-024-04881-8)
Supplement: Supplementary file 10 — Additional file 10: Fig. 8. Hazard ratio (HR) and 95% confidence interval (CI) of contracting and dying in other sepsis, abdominal, urogenital, soft tissue, pneumonia, endocarditis, tuberculosis, and central nervous system infections, adjusted for age (as timescale), sex, marital status, education, smoking status, alcohol consumption and Charlson’s weighted comorbidity index. [file 13054_2024_4881_MOESM10_ESM.pdf]

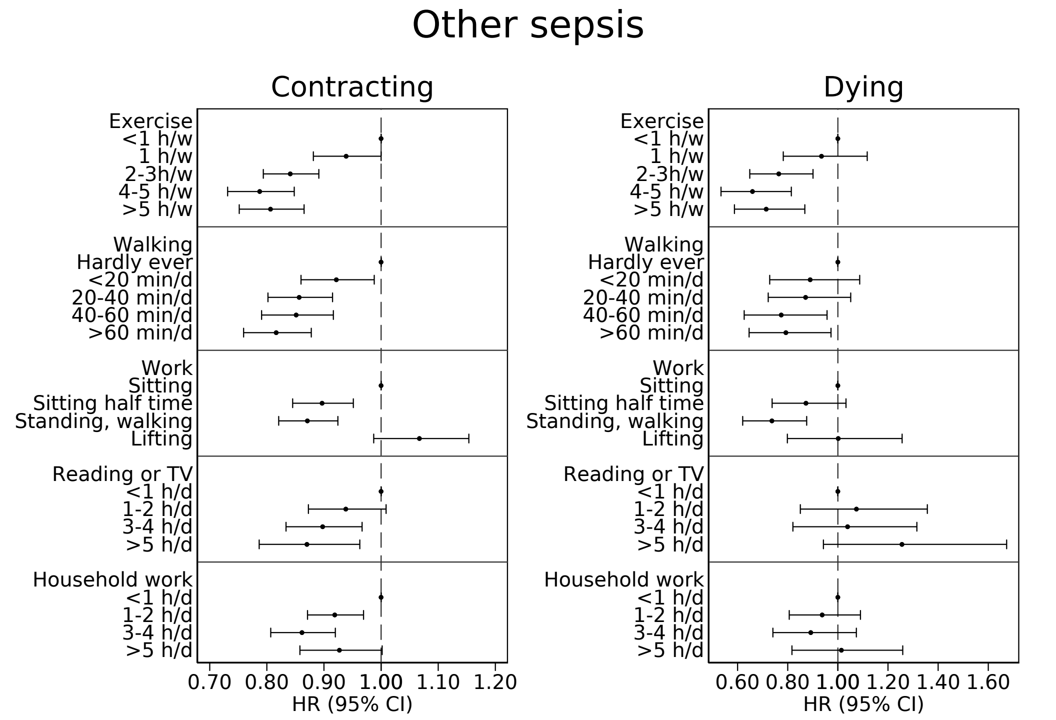
Other sepsis
Contracting
Dying
Exercise
<1 h/w
1 h/w
2-3h/w
4-5 h/w
>5 h/w
Walking
Hardly ever
<20 min/d
20-40 min/d
40-60 min/d
>60 min/d
Work
Sitting
Sitting half time
Standing, walking
Lifting
Reading or TV
<1 h/d
1-2 h/d
3-4 h/d
>5 h/d
Household work
<1 h/d
1-2 h/d
3-4 h/d
>5 h/d
0.70 0.80 0.90 1.00 1.10 1.20
0.60 0.80 1.00 1.20 1.40 1.60
HR (95% CI)

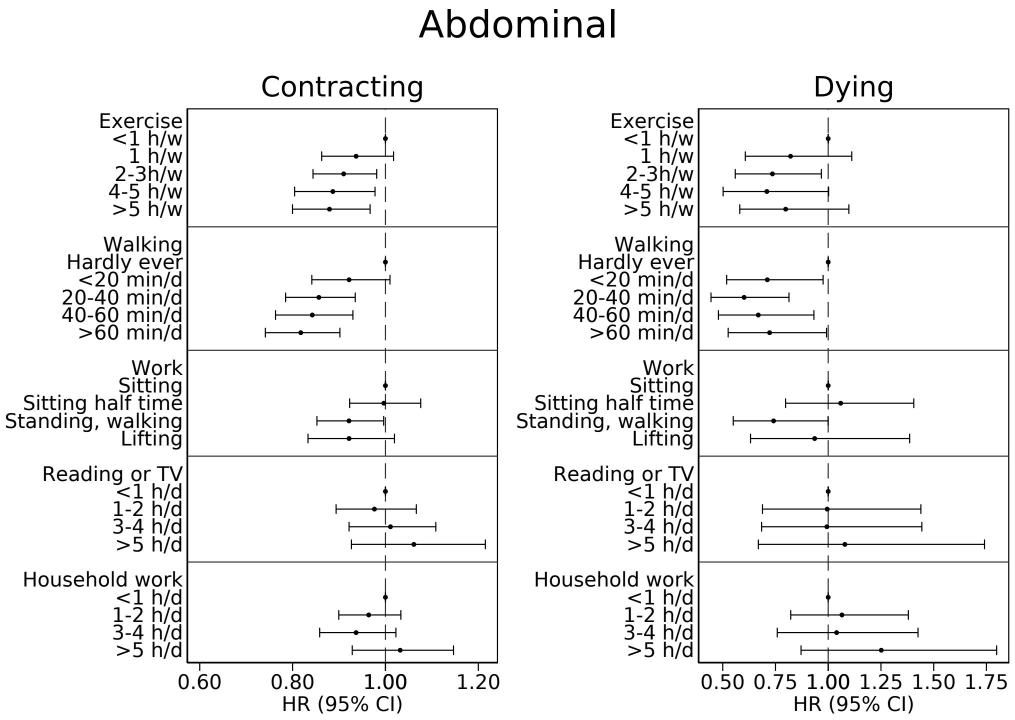
Abdominal
Contracting
Dying
Exercise
<1 h/w
1 h/w
2-3h/w
4-5 h/w
>5 h/w
Walking
Hardly ever
<20 min/d
20-40 min/d
40-60 min/d
>60 min/d
Work
Sitting
Sitting half time
Standing, walking
Lifting
Reading or TV
<1 h/d
1-2 h/d
3-4 h/d
>5 h/d
Household work
<1 h/d
1-2 h/d
3-4 h/d
>5 h/d
0.60
0.80
1.00
1.20
0.50
0.75
1.00
1.25
1.50
1.75
HR (95% CI)

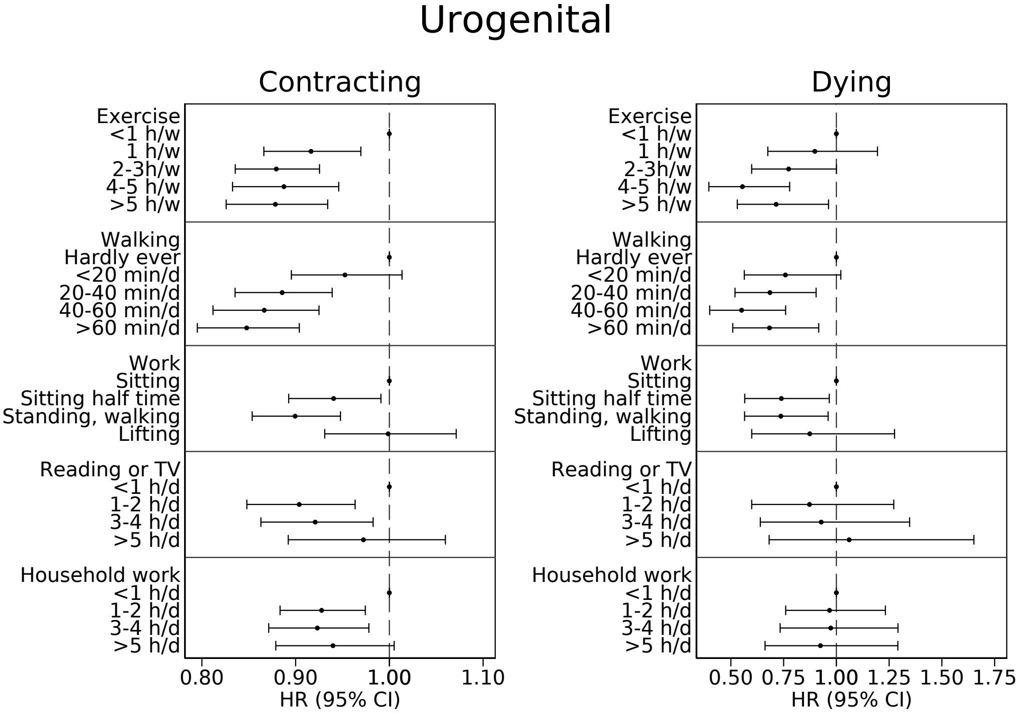
Urogenital
Contracting
Dying
Exercise
<1 h/w
1 h/w
2-3h/w
4-5 h/w
>5 h/w
Walking
Hardly ever
<20 min/d
20-40 min/d
40-60 min/d
>60 min/d
Work
Sitting
Sitting half time
Standing, walking
Lifting
Reading or TV
<1 h/d
1-2 h/d
3-4 h/d
>5 h/d
Household work
<1 h/d
1-2 h/d
3-4 h/d
>5 h/d
0.80
0.90
1.00
1.10
0.50
0.75
1.00
1.25
1.50
1.75
HR (95% CI)

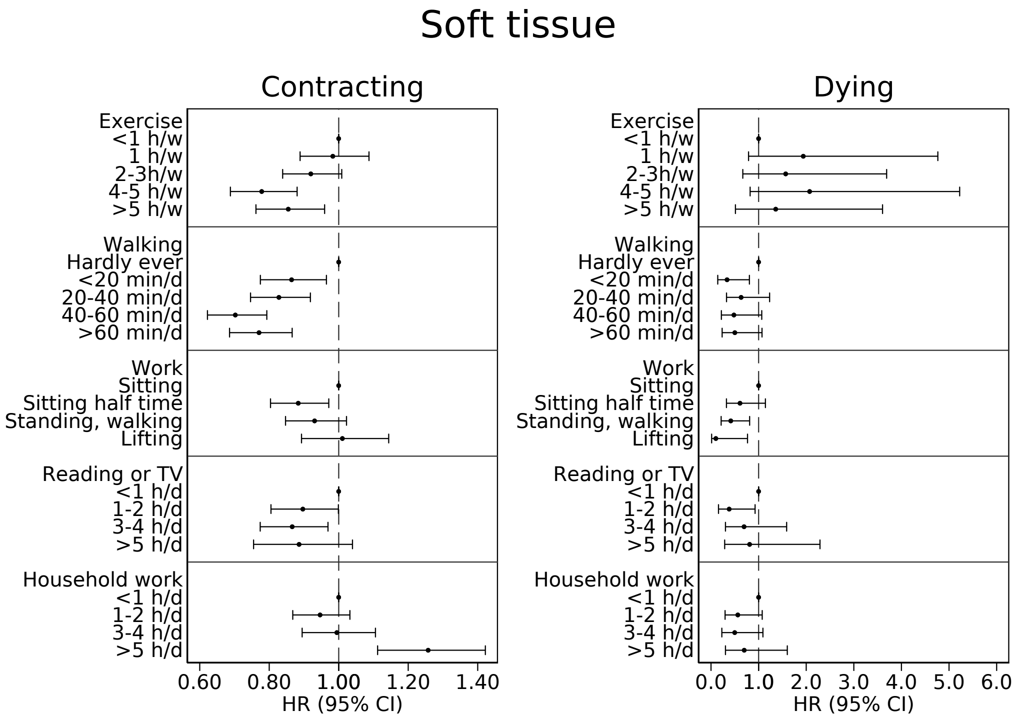

Soft tissue
Contracting
Dying
Exercise
<1 h/w
1 h/w
2-3h/w
4-5 h/w
>5 h/w
Walking
Hardly ever
<20 min/d
20-40 min/d
40-60 min/d
>60 min/d
Work
Sitting
Sitting half time
Standing, walking
Lifting
Reading or TV
<1 h/d
1-2 h/d
3-4 h/d
>5 h/d
Household work
<1 h/d
1-2 h/d
3-4 h/d
>5 h/d
0.60
0.80
1.00
1.20
1.40
HR (95% CI)
0.0
1.0
2.0
3.0
4.0
5.0
6.0
HR (95% CI)

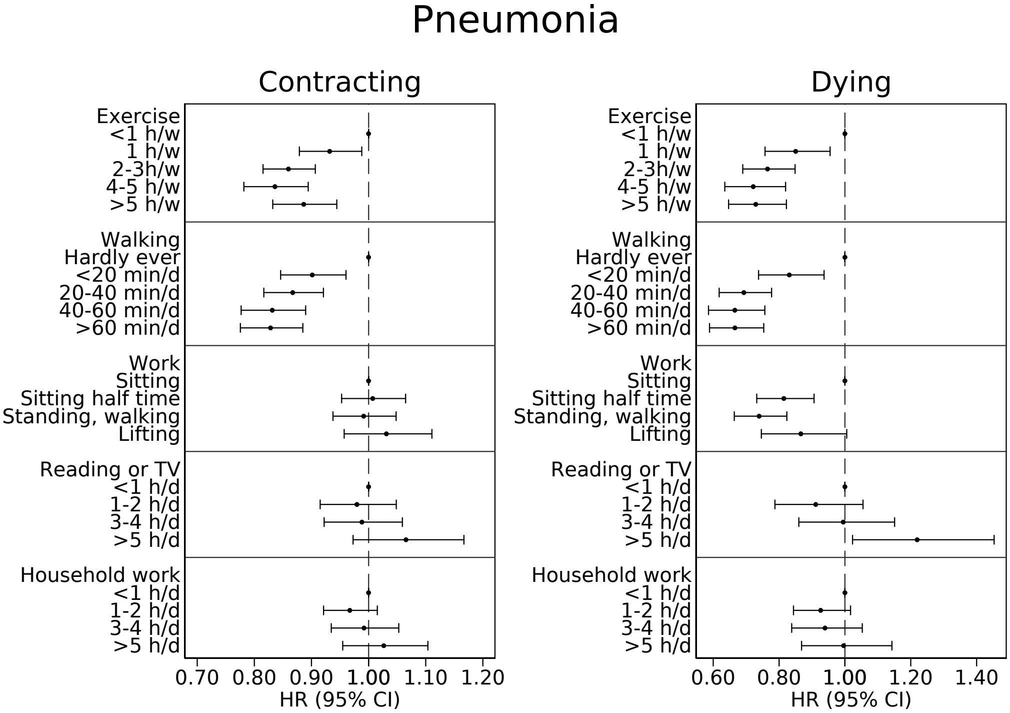

Pneumonia
Contracting
Dying
Exercise
<1 h/w
1 h/w
2-3h/w
4-5 h/w
>5 h/w
Walking
Hardly ever
<20 min/d
20-40 min/d
40-60 min/d
>60 min/d
Work
Sitting
Sitting half time
Standing, walking
Lifting
Reading or TV
<1 h/d
1-2 h/d
3-4 h/d
>5 h/d
Household work
<1 h/d
1-2 h/d
3-4 h/d
>5 h/d
0.70 0.80 0.90 1.00 1.10 1.20
0.60 0.80 1.00 1.20 1.40
HR (95% CI)

Endocarditis

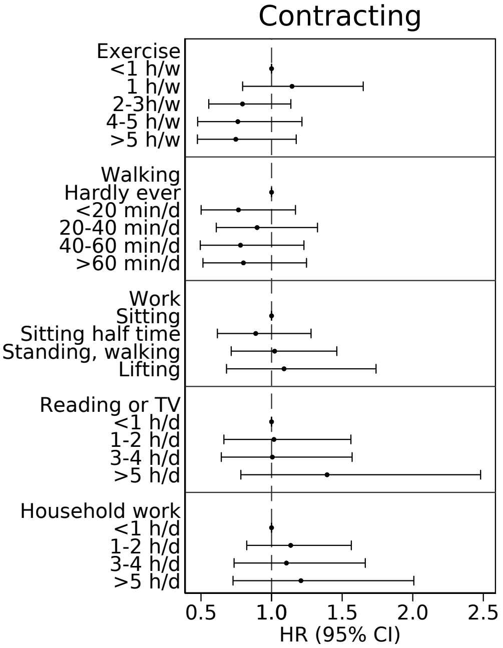

Dying

Too few deaths for analysis (n=27)

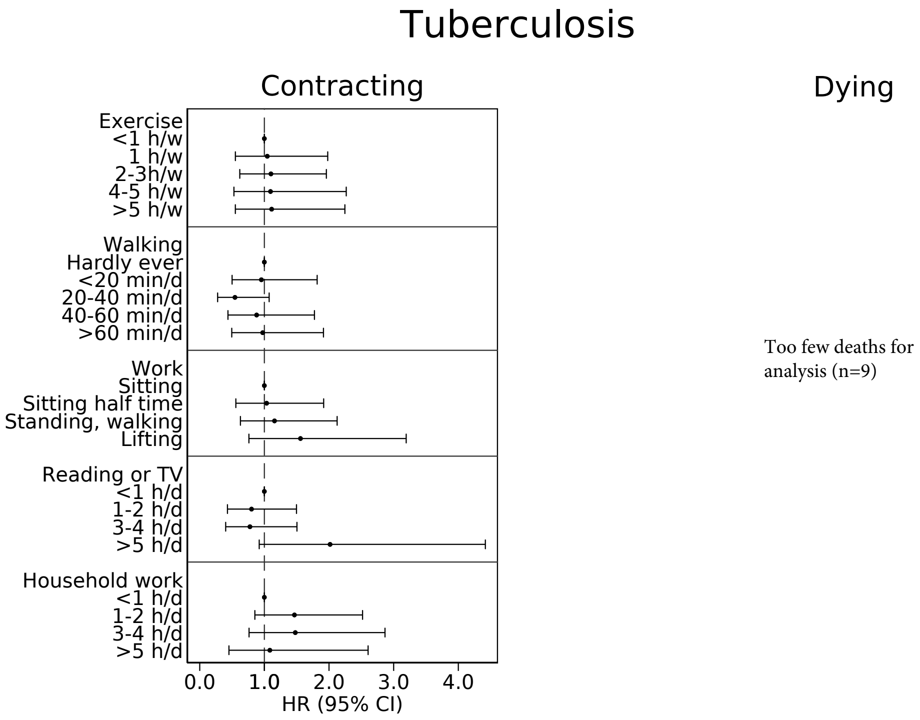
Tuberculosis
Contracting
Dying
Exercise
<1 h/w
1 h/w
2-3h/w
4-5 h/w
>5 h/w
Walking
Hardly ever
<20 min/d
20-40 min/d
40-60 min/d
>60 min/d
Work
Sitting
Sitting half time
Standing, walking
Lifting
Reading or TV
<1 h/d
1-2 h/d
3-4 h/d
>5 h/d
Household work
<1 h/d
1-2 h/d
3-4 h/d
>5 h/d
0.0
1.0
2.0
3.0
4.0
HR (95% CI)
Too few deaths for analysis (n=9)

Central nervous system

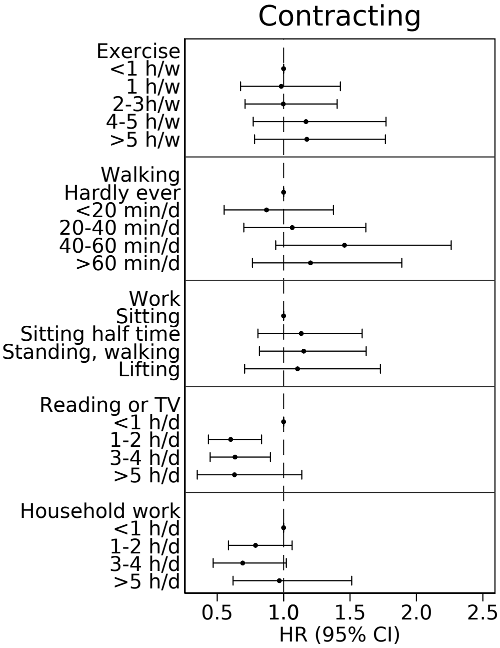

Dying

Too few deaths for analysis (n=18)
